# Supplementary material for: Nanoscale Strontium-Substituted Hydroxyapatite Pastes and Gels for Bone Tissue Regeneration
Source: Nanomaterials (Basel). 2021 Jun 19;11(6):1611. doi: 10.3390/nano11061611 (PMC8235522; doi:10.3390/nano11061611)
Supplement: Supplementary file 1 [file nanomaterials-11-01611-s001.zip › nanomaterials-1254187-SI.pdf]

Supplementary data

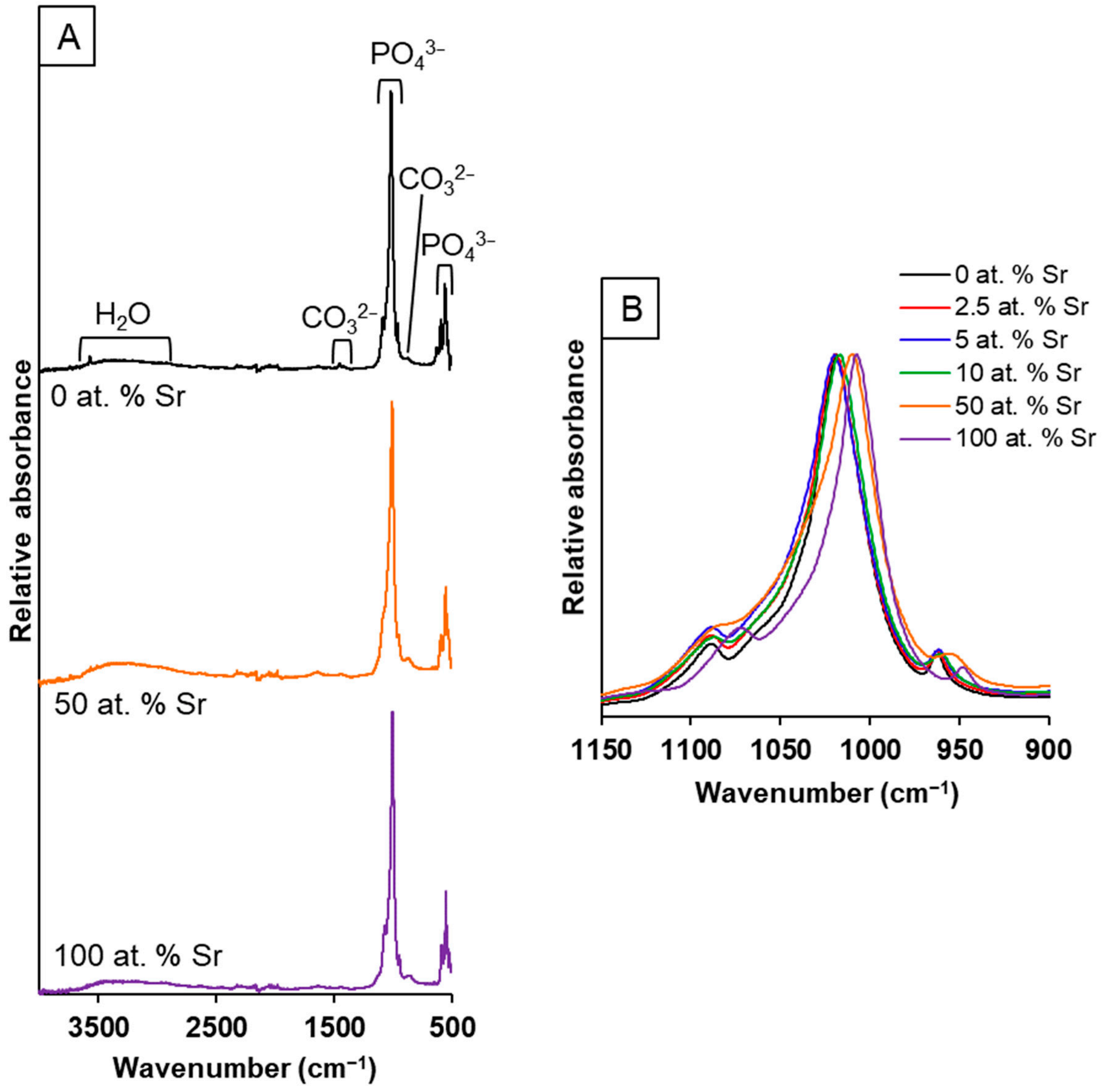

Figure S1. FTIR-ATR spectra of unsintered SrHA powders produced using the sol-gel method: 0, 50 and 100 at.% SrHA (A), and phosphate peaks of 0, 2.5, 5, 10, 50 and 100 at.% SrHA (B).
